# Long-Term Preservation of Human Head and Neck Specimens for Neurosurgical Training: A Technical Note

**DOI:** 10.3390/brainsci15091016

**Published:** 2025-09-20

**Authors:** Francesco Signorelli, Valid Rastegar, Matteo Palermo, Domenico Laino, Fabio Zeoli, Massimiliano Visocchi

**Affiliations:** 1 Department of Neurosurgery, Fondazione Policlinico Universitario “A. Gemelli” IRCCS, 00168 Rome, Italy; 2 Department of Neurosurgery, Università Cattolica del Sacro Cuore, 00168 Rome, Italy; 3 Istituto di Sanità Pubblica, Sezione di Medicina Legale, Università Cattolica del Sacro Cuore, Largo F. Vito 1, 00168 Roma, Italy

**Keywords:** cadaveric preservation, specimen preservation, neurosurgical training, preservation protocol, fresh-frozen specimen, neurosurgery laboratory

## Abstract

**Purpose:** Cadaveric dissection is a cornerstone of neurosurgical education, providing trainees with a realistic 3D understanding of anatomy and a safe environment to practice surgical approaches. A preservation technique was developed that merges the advantages of fresh-frozen and embalmed cadavers, maintaining tissue realism while enhancing durability. This approach preserves flexibility and natural color, improves anatomical detail, and creates a safe, long-lasting model ideal for neurosurgical training. **Methods:** Four specimens were thawed, cannulated, and irrigated before implementing a protocol consisting of low concentration formaldehyde with glycerol and ethanol for extended preservation. The specimens were prepared for both neurosurgery training and educational purposes, and their condition was evaluated with a semi-quantitative scale. Each specimen was evaluated independently by two raters, blinded to the time-point, using a semi-quantitative scale anchored to predefined criteria (0–3 per domain). Inter-rater reliability was calculated using the intraclass correlation coefficient (ICC [2,k]) for continuous scores and Cohen’s κ for categorical agreement. **Results:** Over nine years of intermittent use, the specimens remained in good condition: tissues retained sufficient softness for dissection, injected vessels stayed vivid in color, and no foul odor or microbial growth was observed. The evaluation employed a semi-quantitative scale, with results ranging from 11/14 to 14/14. The mean values demonstrate stable tissue quality over time, with only minor variations in color and perfusion. The inter-rater reliability was high (ICC = 0.91; κ = 0.88). **Conclusions:** The preservation method leverages the strengths of both fresh-frozen and embalmed models. The results suggest feasibility of long-term reuse, although further quantitative validation is needed.

## 1. Introduction

Cadaveric dissections play a pivotal role in the training of surgeons worldwide, especially when approaching complex anatomic regions such as the skull base and the craniovertebral junction.

Anatomic dissections provide trainees with a realistic 3D understanding of anatomy and a safe environment to practice surgical approaches. In particular, the availability of ready-to-use high quality specimens (i.e., cadavers that are not chemically embalmed but preserved by freezing soon after death) is crucial. Particularly, fresh-frozen human specimens offer superior tissue realism compared to formalin-fixed cadavers. Fresh-frozen tissues retain natural coloration, flexibility, and consistency, closely mimicking in vivo conditions, which is invaluable for simulating delicate neurosurgical procedures [1,2].

However, the use of fresh cadavers is not always possible, especially in some regions immediate access is not feasible due to practical, ethical and legislative constraints. To overcome these challenges, cadaver heads are frequently fresh-frozen and later thawed for dissection. Fresh-frozen specimens provide much of the fidelity of fresh tissue while allowing flexible timing for labs and reducing biohazard risks as freezing can reduce bacterial activity. The main limitation is that once thawed, unpreserved tissues will decompose rapidly if not stabilized. Thus, a preservation protocol that maintains tissue quality over time is essential to enable extended use of a specimen for surgical training sessions, especially in cases where, for educational reasons or due to limited resources, frequent reuse of the same specimen is foreseen [3].

Current preservation solutions are commonly obtained by mixing formaldehyde and other chemicals. However, the latter has many disadvantages, including carcinogenicity, tendency to make tissues stiff, and irritation of the skin, eyes, and nasal tissue of the handlers [2].

Differently, traditional formaldehyde-based embalming (10% formalin) prevents decay and allows long-term storage, but at the cost of severe tissue stiffening, color loss, and exposure to toxic fumes [4]. Formalin fixation can compromise vessel patency and neural tissue pliability, diminishing realistic neurosurgical simulation.

Recently, several authors proposed alternative protocols: the Modified Thiel method with validated quantitative scoring [5], the Modified Larssen solution as a low-cost option [6], and formalin-free embalming with N-vinyl-2-pyrrolidone [6,7]. Despite these advances, evidence on long-term preservation exceeding two years remains scarce [8].

In the anatomical dissection laboratory of the Fondazione Policlinico A. Gemelli in Rome, which is primarily dedicated to approaches to the cranio-cervical junction, a preservation protocol is employed for head and neck specimens with the following aims:

- Ensure optimal yield of parenchymal, nervous and vascular structures, typical of such complex anatomical region.
- Support the adjunctive use of image-guidance systems, such as the surgical microscope, endoscope, and neuronavigation, to broaden the learning opportunities during anatomical dissections and enhance the spatial orientation of surgeons in training [9].
- Reuse the heads for training and educational/demonstrative purposes during practical exercises for students for university courses and masters.

Herein, we summarize our experience using a modified preservation protocol for human head and neck specimens devoted towards neurosurgical dissection training and present the results of our preserved specimens after a period of 9 years.

## 2. Materials and Methods

The frozen human head and neck specimens were preserved and prepared following a modified version of Sanan et al. protocol [10], first outlined in our previous study by Signorelli et al. [9], and performed at our own Institution starting 2016 [11,12].

The specimens were stored at −20 °C to −30 °C until use, with no mandatory minimum storage time before thawing. The preservative solution was developed by the authors, with similarities to ethanol–glycerol approaches described in the literature. Silicone was chosen for vascular injection due to its proven long-term stability and widespread use in neurosurgical anatomy labs [10].

This protocol was applied to 4 specimens intended for repeated surgical research use. Of those, one was rendered unusable due to significant leakage from the cannulation site the vessel.

The cadaveric head and neck specimens used in this protocol were obtained through a body donation program with informed consent from the donor specifically permitting use for medical education and research. Ethical approval for cadaver use was in place under our institutional guidelines, and the procedure adhered to the Declaration of Helsinki and local laws governing human tissue research. Before preservation, the specimens were screened for infectious diseases as per standard safety protocols (e.g., HIV, hepatitis) to protect personnel. Throughout the preparation, participants wore appropriate personal protective equipment (PPE) including gloves, gowns, eye protection, and respirators when handling chemicals like formaldehyde. All work was conducted in a ventilated laboratory space by taking appropriate biohazard precautions. These measures ensured a respectful and safe use of the donated specimen for educational purposes.

### 2.1. Thawing, Vessels Cannulation and Irrigation

The thawing process was performed at a controlled temperature of 0–4 °C for 72 h to ensure gradual defrosting. The fresh-frozen human head was stored at −20 °C to −30 °C until use. The frozen specimen was transferred to a refrigeration unit at 0–4 °C, where it was allowed to defrost over approximately 72 h. Maintaining a constant near-freezing temperature during thawing helps prevent ice crystal formation and tissue dehydration. This guarantees a uniform warming of the brain, including deeper structures, thus preserving cellular architecture from thermal shock.

After thawing, major vessels were carefully cannulated to allow for irrigation and subsequent perfusion. The major aim of vessels cannulation was to isolate the great vessels: the common carotid arteries, internal jugular veins on each side, and the vertebral arteries. Cannulation was performed using a 30 mL syringe to ensure optimal flow through the vascular network.

Once cannulated, the vascular system was irrigated with cold water to remove residual blood clots, and air emboli that might still be present and cause an obstruction to vessel patency. By the end of this stage, the vascular system was patent and clear. All major arteries and veins demonstrated patency on our specimen, as evidenced by symmetric outflow during flushing with water.

### 2.2. Fixation and Preservation

After vascular irrigation, the specimen underwent chemical fixation to stabilize tissues and prevent decomposition. The fixation protocol was developed to preserve tissue integrity at a level between that of fresh and fully embalmed specimens. First, the head was submerged in a 4% formaldehyde solution for 5–7 days. The head was contained in a sealed container containing roughly 10 L of the fixative, ensuring that the entire specimen was fully immersed. This initial fixation cross-links proteins and halts enzymatic decay. We chose a 4% formaldehyde as this concentration has been shown to both disinfect and preserve, while causing minimal stiffening compared to traditional embalming protocols. The specimen was kept at room temperature (23 °C) for a week to facilitate formalin penetration. We periodically shook the solution to ensure that the fixative reached all internal spaces equally and did not settle.

Following formalin fixation, the specimen was transferred into our solution. The total volume prepared was 8 L, which consisted of the following: 60% water, 20% formaldehyde, 10% glycerol, and 10% ethanol. The head was stored submerged in this solution when not being actively dissected. Each component of the solution serves a purpose: formaldehyde prevents microbial growth and preserves tissue structure; ethanol acts as both a disinfectant and preservative; glycerol keeps tissues moist and pliable, counteracting the rigidity and shrinkage given by the formaldehyde; and water that serves as the solvent and provides volume for submersion. The head was stored at room temperature (or refrigerated at 4 °C for very long-term storage) in a sealed container to prevent evaporation of ethanol and escape of formaldehyde fumes. Over time, the tissues of the specimens remained well-hydrated and only moderately firm.

### 2.3. Perfusion

To facilitate vascular identification during neurosurgical dissection, colored silicone injection of the head’s arterial and venous systems was performed with a low-viscosity, rapid-catalysis silicone. The silicone was prepared in two containers: one with a red-tinted mixture for the arteries and one with a blue-tinted mixture for the veins.

At this stage, the vessels were uniformly filled with colored silicone, without significant gaps, indicating a successful perfusion. While alternative perfusion materials such as latex exist and have certain advantages like lower cost and no need for mixing catalysts, we chose silicone for its widespread use and proven long-term stability in vessels.

### 2.4. Storage of Specimen

Upon completion of the above steps, the preserved specimen was stored for long-term use. For initial curing and fixation post-injection, the head was left in a cool, ventilated area for 24 h to dissipate any remaining formaldehyde and allow the silicone to fully set. Afterwards, the specimen was placed back into the preservation solution inside the sealed container. The head remained submerged in this fluid when not actively being dissected. The container was kept refrigerated at 4 °C between uses to prevent bacterial growth. We periodically checked the specimen and solution about once every 5–6 weeks to monitor for any signs of mold, desiccation, or deterioration of tissue. As ethanol can evaporate over time even in a closed container, additional glycerol was periodically added to the solution to maintain moisture.

### 2.5. Acquisition of Surgical Equipment

One advantage of this protocol is that it can be performed with relatively basic laboratory equipment, making it accessible for in-house implementation. Key equipment and materials used include:

Refrigeration units

A standard freezer (−20 °C) for long-term specimen storage, and a laboratory refrigerator (0–4 °C) for controlled thawing.

Dissection instruments

The instrumentation included microsurgical tools such as scalpels, forceps, scissors, rongeurs, and retractors for tissue dissection, as well as a vacuum aspirator and a high-speed drill.

Cannulation supplies

Scalpels for vessel exposure, hemostats and forceps for isolating vessels, a set of catheters/tubing (Foley catheters 12–18 Fr and various diameter plastic tubes), silk or cotton sutures (0–0 or 2–0) to tie cannulas in place, and syringes or an irrigation pump for flushing.

Preservative chemicals

10% neutral buffered formalin (prepared from commercial 37% formaldehyde stock), glycerol (laboratory or pharmaceutical grade), ethanol (95% denatured ethanol for mixture), and containers for mixing/storing solutions. All chemical handling was performed with fume extraction, either in a fume hood or well-ventilated space due to formaldehyde release.

Silicone injection kit

Silicone rubber compound (a two-part room-temperature vulcanizing silicone kit designed for anatomical injections was used), color pigments (red and blue), mixing cups and stirring sticks, large syringes (50–100 mL) with Luer-lock fittings to attach to cannulas, and clamps to occlude or open vessels as needed during injection.

Storage container

A large, sealable container to hold the specimen in preservative solution; ideally opaque or kept in the dark to prevent algal growth and to reduce light exposure which can degrade some chemicals.

Protective gear

Gloves (nitrile), safety glasses, scrubs, plastic aprons or lab coats, and respirators (especially when working with formalin or during draining of fluids).

### 2.6. Evaluation and Assessment of Cadaveric Specimens After 9 Years

The texture of the parenchyma and the efficiency of preservation were evaluated based on the quality of vascular injection, specific anatomical features such as the lower cranial nerves, cerebellar microvasculature, and parenchymal texture using a 3-point rating scale (1 to 3) for each feature, with a total score out of 14, as detailed in Table 1.

A specimen assessment protocol, adapted from Mignucci-Jiménez et al. [2], was developed to evaluate the cadaveric preparations. The scale consisted of three main categories: parenchymal texture, preservation quality, and injection quality. Parenchymal texture and preservation quality were evaluated at three-year intervals, while injection quality was assessed at baseline. A composite score ranging from 5 (lowest) to 14 (highest) was calculated based on this evaluation framework.

Each specimen was evaluated independently by two raters, blinded to the time-point, using a semi-quantitative scale anchored to predefined criteria (0–3 per domain). Inter-rater reliability was calculated using the intraclass correlation coefficient (ICC [2,k]) for continuous scores and Cohen’s κ for categorical agreement.

## 3. Results

All four specimens (3 Female, 1 Male) were formalin-fixed (72.5 ± 6.1 years), by lab technicians instructed by the neurosurgeons. Three cases were injected with silicon, whereas one was injection-free. In three cases, contrast medium (Iomeron) was injected both after fixation and prior to vascular injection in order to perform a CT scan following a neuronavigation protocol.

Table 2 shows results at 9-year follow-up. Three specimens were rated between 11/14 and 14/14. In these cases, the baseline injection was optimal, mainly due to the patency of the Circle of Willis, the effective cannulation and fixation, and the silicone viscosity and dilution. Nonetheless, parenchymal texture and overall preservation, including structural integrity, tissue quality and odor, remained optimal throughout the years. Slight discoloration was recorded in case #3, due to dye leakage during the injection phase. Case #4 showed significant dye leakage, resulting in ineffective vascular filling and lower overall grading score.

Table 3 summarizes the preservation scores assigned by two independent raters across the three follow-up periods. The mean values demonstrate stable tissue quality over time, with only minor variations in color and perfusion. The inter-rater reliability was high (ICC = 0.91; κ = 0.88).

**Figure 1:**
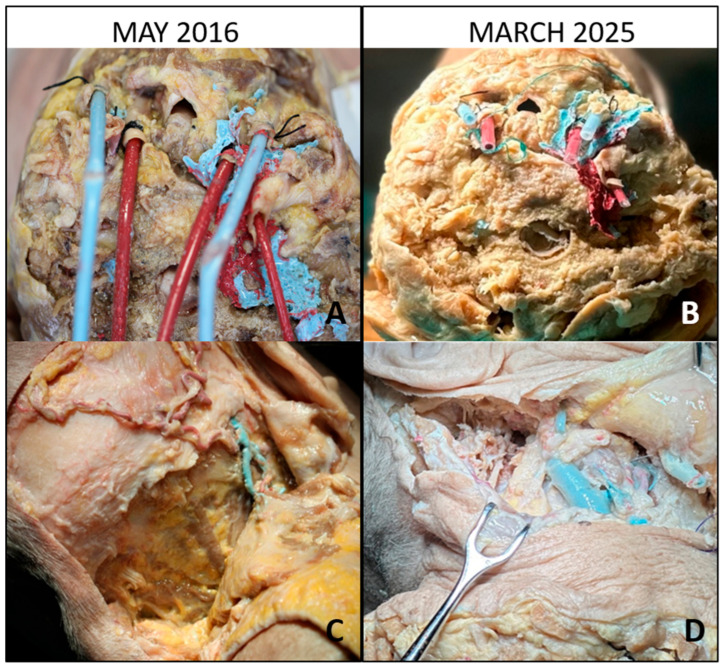
Baseline and long-term evaluation of vascular cannulation and posterolateral exposure. (**A**) Baseline view (May 2016): cannulation of the common carotid arteries and jugular veins with colored silicone perfusion of the vascular system. (**B**) After 9 years (March 2025): preserved patency of the injected vessels, with silicone still visible despite minor discoloration. (**C**) Baseline posterolateral approach: inverted hockey-stick incision with stepwise muscular dissection, exposing the suboccipital triangle. (**D**) After 9 years: the same approach demonstrating satisfactory tissue pliability and preserved anatomical detail of the suboccipital region.

**Figure 2:**
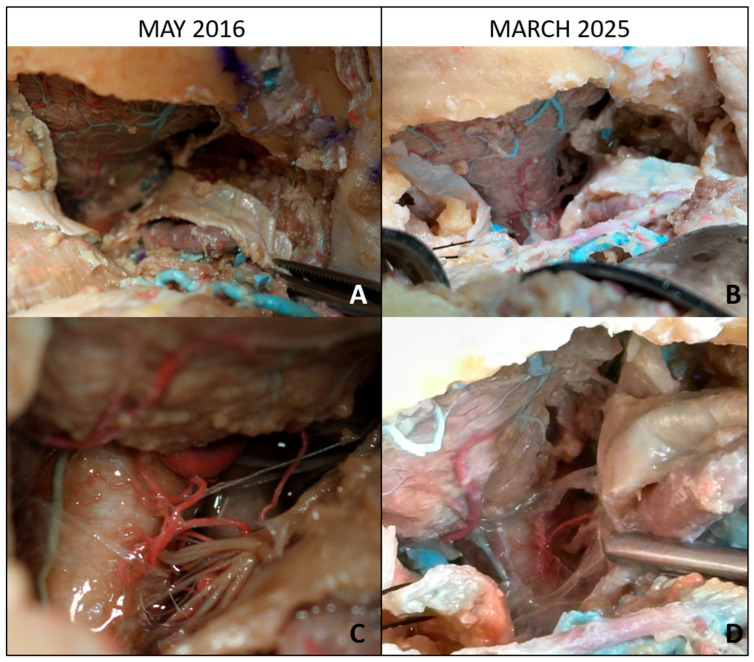
Baseline and long-term evaluation of lateral suboccipital approaches with intradural exposure. (**A**) Baseline view (May 2016): lateral suboccipital craniectomy with partial removal of the posterior arch of C1, exposing the cerebellar surface and its vascularization. (**B**) After 9 years (March 2025): preservation of the operative field with identifiable cerebellar vasculature despite long-term storage. (**C**) Baseline intradural exposure: bulbomedullary junction, lower cranial nerves, brainstem, and posterior inferior cerebellar artery (PICA) clearly visible. (**D**) After 9 years: corresponding intradural field showing satisfactory anatomical preservation of neurovascular structures, though with moderate tissue color changes.

Over 9 years of intermittent use, around thrice per year, the specimens remained in good condition, with the tissues retaining sufficient softness for dissection, the colors of the injected vessels stayed vivid, and no foul odor or microbial growth was observed (Figure 1 and Figure 2). Between training sessions, the specimens were rinsed with fresh water to remove any debris and then returned the specimen to the preservative bath. Wrapping the head in a preservative-soaked cloth before sealing it kept all surfaces moist. The renewal of the embalming solution was performed once a year ($25).

## 4. Discussion

The traditional embalming technique consists of a conservatory solution made with 10% formalin (4% formaldehyde) and additional chemicals (methanol, phenol, etc.). While these specimens find common applications in anatomy labs due to their longevity, they are unsuitable for neurosurgical training because the formalin preparation makes the tissue rigid and discolored. Therefore, the brain tissue becomes firm and may not be easily retracted or dissected in a life-like manner (Table 4).

Some improvements have been made by using lower concentrations. For example, O’Sullivan et al. [16] outlined using <5% formaldehyde solutions could preserve the specimen while reducing stiffness, but even “lightly embalmed” specimens are less flexible than fresh ones. Our protocol uses formaldehyde in a reduced, targeted way, both for specimen fixation and for soaking solution; accounting for 20% of the final soaking solution to limit the aforementioned issues. We observed our specimen’s tissues were less rigid than a typical formalin cadaver, confirming that limiting formaldehyde exposure preserves more natural consistency, preserving pliability. When compared to Ray et al. [7], Bilge & Celik [5], Haizuka et al. [17], and Nagase et al. [6], the present method is less innovative chemically but unique in demonstrating reuse for over nine years. The assessment scale applied here is semi-quantitative and subjective; therefore, the findings should be considered preliminary. Future studies should incorporate validated scoring frameworks, quantitative metrics (e.g., elasticity tests, radiological imaging), and inter-observer reliability to strengthen reproducibility and reduce bias.

A modern alternative to formalin is embalming with high concentrations of ethanol, often with additives like glycerin and small amounts of formaldehyde or other agents. Ethanol acts as a fixative and antimicrobial agent, and glycerin prevents excessive drying. Studies by Coleman et al. [15] and Sanan et al. [10], have shown that cadavers embalmed with predominantly ethanol-glycerin can remain soft and flexible, with reduced toxic odor. Sanan’s method for neurosurgical specimens comprising a 66% ethyl alcohol solution for long-term preservation, reporting good results over two years [10]. Our preservative solution can be seen as a variant of this approach: it contains a significant fraction of ethanol and glycerol, mixed with low concentration of formaldehyde. The presence of formaldehyde in our solution provides additional cross-linking for durability, but the concentration is lower than standard embalming fluid.

Differently, some training labs simply thaw frozen cadavers shortly before use, with minimal or no chemical preservation yielding an experience very close to a fresh cadaver. The clear benefit to this approach is tissue realism as freezing alone does not drastically alter mechanical properties of the tissue. However, many institutions, including our own, do not use fresh-frozen specimens due to their rapid decay. This leads to the need for more frequent replacements, which can be costly and depends on the availability of specimens.

The Thiel method is a widely used embalming, it consists of a multi-component solution, including salts, boric acid, nitrates, ethylene glycol, and very low formaldehyde, to embalm whole cadavers. Thiel-embalmed cadavers are known for their remarkably life-like tissue appearance. However, Thiel’s method has certain drawbacks. For example, it is complicated, costly, and requires careful preparation of multiple solutions and large volumes of chemicals [13,18,19,20].

Hammer et al. compared various preservation techniques and emphasized the importance of balancing cost, technical complexity, and tissue quality, which aligns with the goals of our protocol [14]. Therefore, for resource limited laboratories, our protocol can offer a compromise in terms of cost and require less technical expertise while resulting in adequate preservation of the specimen.

The preserved specimens have been utilized in our dissection laboratory for various training exercises, including university courses and master’s programs focusing on the craniocervical junction [12]. Trainees noted that the tactile feedback was superior to that of formalin-fixed specimens they had used previously. Realistic responses of tissue to manipulation more akin to a live scenario could also be observed.

A major takeaway from our analysis is the need to regularly change the immersion solution to avoid compromising both structural and olfactory qualities. This is because stagnant solutions can lead to microbial growth, oxidation, and desiccation of tissues undermining the overall quality of the specimen. This simple step plays a foundational role in maintaining tissue hydration and preventing the breakdown of vascular and neural structures.

Additionally, during dissection phases, it is crucial to frequently rinse the specimen with the physiological solution. This practice helps prevent both the pliability and authenticity of the tissues, while also improving handling, thus enhancing the dissection experience.

Exposure to light is another often-overlooked factor. Direct or prolonged light contact can accelerate tissue drying and lead to irreversible changes to the texture. Therefore, specimens should be stored in darkness when not in use, and dissection lights should be managed carefully to reduce damage.

Overall, a successful long-term cadaveric preservation is not merely the result of good initial preparation, but rather the result of continuous attentive care while storage, and careful intraoperative handling during dissection. The integration of two independent evaluators and the demonstration of strong inter-rater reliability indicate that, despite the semi-quantitative nature of the scale employed, the results are consistent across observers. This approach may provide a more reproducible framework for evaluating long-term cadaver preservation protocols. Nevertheless, the present study is limited by the small number of cases examined and the absence of objective physical or histological measurements, which restricts the generalizability and depth of the conclusions. Future research with larger cohorts and more rigorous, quantitative assessments is needed to validate and extend these findings.

## 5. Conclusions

Our preservation method attempts to leverage the strengths of both fresh-frozen and embalmed models. By freezing and slow thawing, we maintained tissues close to their natural state; by flushing and lightly fixing, we postponed decay; by adding glycerol/ethanol, we retained flexibility and minimized toxins; and by injecting silicone, we markedly enhanced the anatomical detail visible to the trainee. The outcome is a specimen that we found to be highly effective for neurosurgical training, allowing repeated positioning, drilling, and dissection with an anatomical realism approaching that of a fresh cadaver. Overall, the findings suggest that this approach may be particularly suitable for neurosurgical training programs, although we acknowledge that future work should focus on incorporating more standardized and quantitative assessment metrics to enhance the reproducibility and scientific robustness of our findings.

## Figures and Tables

**Table 1:** Preservation total assessment scale for evaluating cadaveric head specimens.

Parenchyma	1	2	3	Total
Texture of parenchyma	Too soft or hard to be usable	Too soft or hard but usable	Similar to brain parenchyma	3
**Texture of parenchyma total score**				3
**Injection**	1	2	3	**Total**
Indirect Injection quality	Leakage that makes region of interest unusable	Dye leakage; structure no longer visualized	No dye leakage; structure exquisitely visualized	3
**Injection total score**				**3**
**Preservation**	1	2	3	**Total**
Structural integrity	Preservation of vessels and nerves unsatisfactory	Preservation of vessels and nerves satisfactory	–	2
Tissue quality	Tissues destroyed	Presence of oxidation, desiccation, or discoloration	Parenchyma preservation satisfactory	3
Smell	Sample unusable because of offensive odor	Sample usable despite offensive odor	No offensive odor	3
**Preservation total assessment score**				8 Total score______

**Table 2:** Specimen Assessment Scale after 3, 6 and 9 years.

After 3 Years	Parenchyma	Injection	Preservation	Total
#1	3	3	8	14/14
#2	3	3	8	14/14
#3	3	2	6	11/14
#4	2	1	4	7/14
**After 6 Years**	**Parenchyma**	**Injection**	**Preservation**	**Total**
#1	3	3	8	14/14
#2	3	3	8	14/14
#3	3	2	6	11/14
#4	2	1	4	7/14
**After** **9 Years**	**Parenchyma**	**Injection**	**Preservation**	**Total**
#1	3	3	8	14/14
#2	3	3	8	14/14
#3	3	2	5	10/14
#4	2	1	3	6/14

**Table 3:** Specimen Assessment Scale with Inter-Rater Reliability.

Time-Point	Specimen	Rater 1 Total (0–14)	Rater 2 Total (0–14)	Mean Score	Notes	Original Score
3 years	#1	14	14	14	Optimal perfusion	14/14
3 years	#2	14	14	14	Optimal perfusion	14/14
3 years	#3	11	12	11.5	Slight discoloration	11/14
3 years	#4	7	8	7.5	Leakage during injection	7/14
6 years	#1	14	14	14	Optimal perfusion	14/14
6 years	#2	14	13	13.5	Preserved pliability	14/14
6 years	#3	11	11	11	Slight discoloration	11/14
6 years	#4	7	7	7	Leakage during injection	7/14
9 years	#1	14	13	13.5	Optimal perfusion	14/14
9 years	#2	14	14	14	Preserved pliability	14/14
9 years	#3	10	11	10.5	Discoloration increased	10/14
9 years	#4	6	7	6.5	Leakage during injection	6/14

Inter-rater reliability: ICC (2,k) = 0.91 (95% CI 0.75–0.98). Cohen’s κ = 0.88 (95% CI 0.70–0.97).

**Table 4:** Comparative overview of preservation methods for anatomical specimens.

Method	Main Composition	Advantages	Disadvantages	Key References
Traditional Formalin Embalming	10% formalin (4% formaldehyde), often with methanol, phenol	Long-term preservation; widely available; low cost	Severe tissue stiffening, discoloration, toxic fumes; poor for surgical simulation	Brenner 2014 [4]
Fresh-Frozen	No chemical additives; frozen at –20 °C	Preserves near-natural tissue color, flexibility, and consistency; best for realism	Limited storage; rapid decomposition after thawing; high biosafety needs	Jansen 2020 [3]
Modified Thiel	Multi-component: salts, boric acid, nitrates, ethylene glycol, very low formaldehyde	Excellent color, flexibility, and life-like appearance; validated scoring systems	High cost; technically complex; requires large volumes of chemicals	Thiel 1992 [13]; Hammer 2022 [14]
Modified Larssen Solution	Ethanol–glycerol base with low formaldehyde	Cost-effective; tissues softer than formalin; reduced odor	Shorter durability vs. Thiel; less validated for long-term use	Bilge & Celik 2017 [5]
Ethanol–Glycerol Fixation	Ethanol (60–70%), glycerol, with small formaldehyde or thymol	Flexible tissue; reduced toxic odor; relatively simple to prepare	Still less realistic than fresh-frozen; durability uncertain	Coleman 1998 [15]; Sanan 1999 [10]
*N*-vinyl-2-pyrrolidone (Formalin-free)	*N*-vinyl-2-pyrrolidone–based solution	Preserves flexibility; avoids formaldehyde toxicity	Limited availability; less common experience worldwide	Nagase 2022 [6]
Modified Protocol (Current Study)	Stepwise: low conc. formalin fixation → ethanol–glycerol–formaldehyde mix → silicone vascular injection	Balance of flexibility and long-term durability; cost-effective; easy to implement with basic lab setup; validated over 9 years	Semi-quantitative evaluation only; limited cases; lacks histological validation	Present study

## Data Availability

The original contributions presented in this study are included in the article. Further inquiries can be directed to the corresponding author.
